# Recent structural insights into the mechanism of ClpP protease regulation by AAA+ chaperones and small molecules

**DOI:** 10.1016/j.jbc.2022.101781

**Published:** 2022-03-02

**Authors:** Mark F. Mabanglo, Walid A. Houry

**Affiliations:** 1 Department of Biochemistry, University of Toronto, Toronto, Ontario, Canada; 2 Department of Chemistry, University of Toronto, Toronto, Ontario, Canada

**Keywords:** ClpP, ATP-dependent protease, protein degradation, dysregulation of protein activity, AAA+ chaperones, small-molecule activator, allostery, agonist, AAA+, ATPase associated with diverse cellular activities, ACP, activator of self-compartmentalizing proteases, ADEP, acyldepsipeptide, ADP, adenosine diphosphate, ATP/ATPγS, adenosine triphosphate/adenosine 5′-O-(3-thio) triphosphate, BsClpP, *Bacillus subtilis* ClpP protease, Bz-LL, benzoyl-leucyl-leucine, ClpP, caseinolytic protease P, CryoEM, cryoelectron microscopy, EcClpP, *Escherichia coli* ClpP protease, FtsZ, filamenting temperature-sensitive mutant Z, GFP, green fluorescent protein, Hsp104, heat shock protein 104 kDa, IGF, isoleucine-glycine-phenylalanine, LmClpP, *Listeria monocytogenes* ClpP protease, MtClpP, *Mycobacterium tuberculosis* ClpP protease, MWC, Monod–Wyman–Changeux, NmClpP, *Neisseria meningitidis* ClpP protease, NMR, nuclear magnetic resonance, OS, oligomerization sensor, PA/LS, probabilistic anticlockwise/long step, PAN, proteasome-activating nucleotidase, QAT, glutamine-alanine-threonine, QFS, glutamine-phenylalanine-serine, QXT, glutamine-any amino acid X-threonine, RKH, arginine-lysine-histidine, RNA, ribonucleic acid, SaClpP, *Staphylococcus aureus* ClpP protease, SAXS, small-angle X-ray scattering, SC/2R, sequential clockwise/2-residue (step), SMAC, small molecule activated ClpP, TROSY, transverse relaxation-optimized spectroscopy, ZBD, zinc-binding domain, Z-GLF-CMK, benzyloxycarbonyl-Gly-Leu-Phe-chloromethyl ketone

## Abstract

ClpP is a highly conserved serine protease that is a critical enzyme in maintaining protein homeostasis and is an important drug target in pathogenic bacteria and various cancers. In its functional form, ClpP is a self-compartmentalizing protease composed of two stacked heptameric rings that allow protein degradation to occur within the catalytic chamber. ATPase chaperones such as ClpX and ClpA are hexameric ATPases that form larger complexes with ClpP and are responsible for the selection and unfolding of protein substrates prior to their degradation by ClpP. Although individual structures of ClpP and ATPase chaperones have offered mechanistic insights into their function and regulation, their structures together as a complex have only been recently determined to high resolution. Here, we discuss the cryoelectron microscopy structures of ClpP-ATPase complexes and describe findings previously inaccessible from individual Clp structures, including how a hexameric ATPase and a tetradecameric ClpP protease work together in a functional complex. We then discuss the consensus mechanism for substrate unfolding and translocation derived from these structures, consider alternative mechanisms, and present their strengths and limitations. Finally, new insights into the allosteric control of ClpP gained from studies using small molecules and gain or loss-of-function mutations are explored. Overall, this review aims to underscore the multilayered regulation of ClpP that may present novel ideas for structure-based drug design.

Proteostasis is the dynamic regulation of the proteome to suit cellular requirements and is maintained by the integrated and competing pathways of protein biogenesis, chaperone-assisted folding, trafficking, and degradation (1, 2). Maintaining proteostasis is critical for normal cell development, healthy aging, and robust stress response to environmental factors including assault from pathogens (3, 4). On the other hand, loss of proteostasis due to protein misfolding and aggregation causes various neurodegenerative diseases and other proteopathies including Alzheimer’s, Parkinson’s, Huntington’s, and Creutzfeldt–Jakob diseases (1).

Proteolytic systems function in proteostasis by identifying and degrading misfolded, mistranslated, or excess proteins, using proteases and *A*TPases *a*ssociated with diverse cellular *a*ctivities (AAA+) that typically act in complex with the proteases (2, 5, 6, 7, 8, 9, 10). The caseinolytic protease P (ClpP) is one such protease that is highly conserved in both prokaryotes and in the mitochondria and plastids of eukaryotes (11, 12, 13, 14, 15). ClpP is a serine protease that assembles into heptameric rings, which then form a double ring, tetradecameric structure containing a chamber where proteolysis occurs (Fig. 1*A*). Peptide bond cleavage is catalyzed by the canonical serine–histidine–aspartate catalytic triad present in each ClpP subunit (Fig. 1*B*). Tandem cleavage events within the ClpP chamber contribute to the timely hydrolysis of protein targets (10). In some organisms such as Mycobacteria, *Listeria*, *Chlamydia*, and Arabidopsis, more than one ClpP isoform exist and have different properties and functions (15, 16, 17, 18, 19, 20, 21, 22).

**Figure 1:**
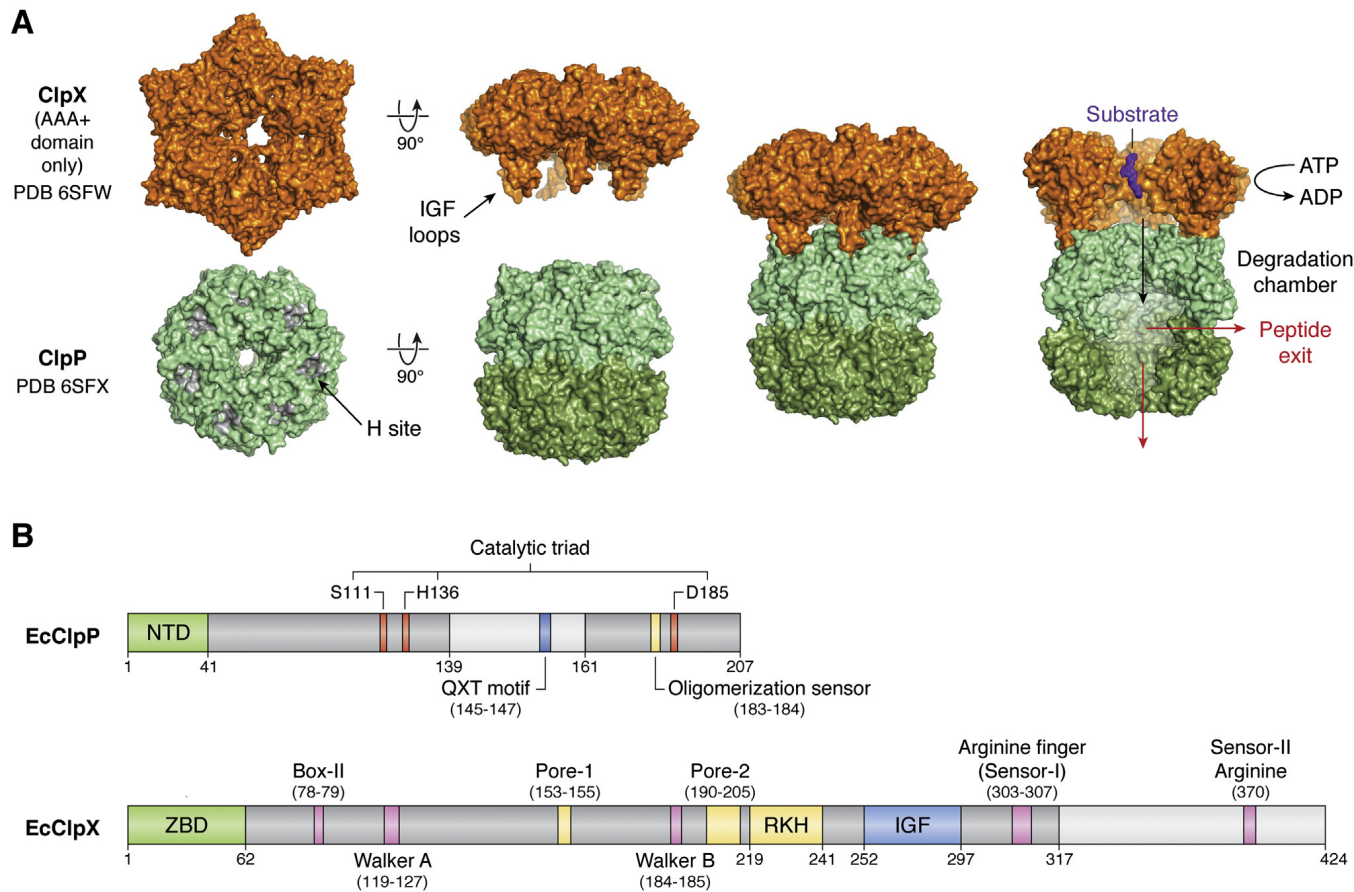
**Domain organization of a representative ClpP-ATPase complex.***A*, structure of representative ClpX and ClpP complexes. ClpX forms a hexameric ring for the recognition and unfolding of protein substrates. Recognition is dependent on N-terminal zinc-binding domains (ZBD) that can dimerize in the complex but is not shown in the figure. ClpP forms a heptameric ring that can further assemble into a double-ring tetradecameric cylinder where proteolytic degradation of substrate occurs. Hydrophobic (H) sites on ClpP are formed by two adjacent ClpP subunits (*gray patches*) and serve as docking sites for conserved IGF loops of ClpX. Complexation of ClpX and ClpP enables ATP-dependent unfolding and degradation of protein substrate shown as purple spheres in *right* most image. In this image, two front subunits of ClpX and four front subunits of ClpP have been removed to show the interior of the ClpXP complex, where the unfolded substrate is threaded and degraded. Degraded peptides exit the ClpP chamber *via* transient side pores and/or the axial pore of an uncapped ClpP ring (*red arrows*). The PDB IDs for the structures are 6SFW (*Listeria monocytogenes* ClpX, LmClpX) and 6SFX (LmClpP1P2). The two homoheptameric rings of LmClpP1P2 are colored *light green* (LmClpP2) and *dark green* (LmClpP1). LmClpX can bind to either LmClpP1 or LmClpP2. *B*, bar diagram of the *E. coli* ClpP (EcClpP) and ClpX (EcClpX) sequences showing conserved domains important for function. EcClpP has three main domains: the N-terminal motif (*green box*) important for EcClpX interactions and for axial pore regulation, the core domain (*dark gray boxes*) that contains the Ser-His-Asp catalytic triad (*red boxes*) and the oligomerization sensor residues (*yellow box*), and the handle domain (*light gray box*, sandwiched by core domain sequences) important for oligomerization of two heptameric rings. The QXT motif (*blue box*) essential for stabilizing the interface between two EcClpP heptamers is also found in the handle domain. EcClpX consists of three main domains: the N-terminal zinc binding domain (ZBD, *green box*) important for substrate recognition, and the large (*dark gray box*) and small (*light gray box*) AAA+ domains that together form the ATP hydrolysis and motor modules. The large AAA+ domain contains the sequences for ATP binding (Box-II, Walker A, Walker B, and Sensor-I Arginine finger, all *pink boxes*), substrate recognition, unfolding, and translocation (pore-1, pore-2 and RKH loops - all *yellow boxes*), and the IGF loop for binding EcClpP (*blue box*). The small AAA+ domain (light gray box) contains the Sensor-II Arginine residue (*pink box*) for ATP binding. Numbers indicate residue positions. ClpP, caseinolytic protease P; EcClpP, *Escherichia coli* ClpP protease; LmClpP, *Listeria monocytogenes* ClpP protease.

Many crystal structures of ClpP from different species have been determined and show an essentially conserved structure composed of the N-terminal loop, the core domain, and the handle region (12). The flexible N-terminal loops can form ordered structures and thus regulate axial pore opening. The core domain contains the catalytic triad and the oligomerization sensor. The latter is important in stabilizing the tetradecameric chamber, which is assembled primarily through the interdigitation of handle domains (Fig. 1*B*). Additionally, adjacent ClpP subunits in the complex form deep, hydrophobic binding clefts (H sites) on the apical surface of ClpP that serve as docking sites for the ATPase (Fig. 1*A*).

ClpP is generally regulated by one or more cognate ATPase chaperones, such as ClpX, ClpA, and ClpC, through direct binding to form a larger complex (9). These ATPase chaperones are formed by six identical subunits, each one containing one or more AAA+ domains that function in ATP binding and hydrolysis (Fig. 1, *A* and *B*). ClpA/C/X chaperones recognize proteins that display specific degrons (23, 24). This recognition is sometimes mediated by protein adaptors and is further regulated by antiadaptors (25, 26, 27, 28, 29, 30, 31, 32). Degrons are short, generally N- or C-terminal amino acid sequences that are either present in proteins or added to proteins during translation to signal rapid protein turnover (33, 34, 35, 36). As ATPases, ClpA/C/X use conformational changes arising from ATP binding and hydrolysis to generate the power strokes needed to unfold substrates and push them into the ClpP chamber for degradation (Fig. 1*A*) (9).

Each ClpX subunit contains a family-specific zinc-binding domain (ZBD) and an AAA+ module that is further subdivided into large and small AAA+ domains (Fig. 1*B*) (10, 37). The ZBD folds independently of the AAA+ module and can dimerize when expressed in isolation (24, 38). Based on this observation, ClpX can be considered as a trimer of dimers (Fig. 1*A*). The ZBDs are flexibly linked to the AAA+ ring of ClpX and are essential for substrate recognition and interactions with protein adaptors. When viewed from the top of the ClpX hexamer (ClpP below ClpX), the AAA+ modules form a right-handed spiral, in which the small AAA+ domain of one subunit packs against the large AAA+ domain of a clockwise subunit. This interaction buries approximately 2000 Å of surface area and is thus mainly responsible for intersubunit interactions that form the hexamer. The large and small AAA+ domains contribute residues that form the nucleotide binding clefts having conserved motifs (Walker A, Walker B, Sensor I and II arginines) found in each domain (Fig. 1*B*). Rotation between the large and small domains around a short hinge region enables the conformational changes necessary for nucleotide exchange. Moreover, ATP binding but not hydrolysis is needed for ClpX to productively bind ClpP and some substrates (39, 40, 41, 42, 43).

Due to their indispensable roles in cellular proteostasis, ClpP-ATPase complexes have been established in many studies as an important target in antibiotic and anticancer drug discovery (44, 45, 46, 47, 48). In many bacterial pathogens, ClpXP is essential for virulence and stress response regulation (44, 49). In the human mitochondria, the ClpXP substrate pool includes proteins involved in the Krebs cycle, oxidative phosphorylation, mitochondrial translation, and fatty acid and amino acid metabolic pathways (45). Mammalian ClpXP is also involved in the mitochondrial unfolded protein response and in heme biosynthesis (5, 45, 50, 51, 52, 53, 54, 55). In many types of human cancers, ClpP is overexpressed and is required to sustain oncogenesis and tumor metastasis (56, 57, 58).

In recent years, various compounds targeting the ClpP-ATPase complex have been investigated. For ClpP, acyldepsipeptides (ADEPs), activators of compartmentalized proteases (ACPs), and imipridones act as agonists that can dysregulate ClpP, causing death in pathogenic bacteria and in cancer cells (59, 60, 61, 62, 63). Phenyl esters and β-lactones have been developed as specific ClpP inhibitors (64, 65). Small molecules that target ClpX and ClpC include cyclomarin A, rufomycin I, ecumicin, and lassomycin (66, 67, 68, 69, 70, 71, 72).

The development of the above small-molecule modulators has been aided by many X-ray crystal structures of ClpX and of ClpP from both bacteria and human (62, 63, 68, 73, 74, 75, 76, 77, 78, 79, 80, 81, 82, 83). Structures of the ClpP-ATPase complex, however, had been difficult to determine by X-ray diffraction methods owing to the flexibility of the components’ interactions but which underlies their coupled unfoldase and protease functions (84, 85). ClpX is inherently more flexible than ClpP, such that its crystal structure was solved only after genetic manipulation to assemble a covalently linked pseudohexamer lacking the N-terminal ZBD (86). However, this mutant ClpX structure does not have the same spiral topology as related disaggregases/ATPases and might not be physiologically relevant, limiting inference of the enzyme’s precise mechanism (87, 88). To overcome this barrier, insights into ClpP regulation by ClpX were extrapolated from studies involving small-molecule agonists that mimic the activating effects of ClpX binding (76, 83, 89, 90, 91). These small molecules bind to H sites on the apical surface of ClpP where the conserved IGF loops of ATPases also bind (76) (Fig. 1*A*). In doing so, the small molecules induce ClpP activation but without the selectivity imposed by ClpX since they displace the ATPase, leading to ClpP dysregulation allowing the protease to unspecifically cleave proteins that enter the catalytic chamber, which is the basis of the antibiotic and anticancer effects of these compounds (59, 85, 92). Many of these small-molecule agonists have very high affinities to ClpP and cause rapid dissociation of ClpX at substoichiometric concentrations. Thus, after forming a complex with ClpP, the resulting small molecule activated complex (SMAC) does not completely recapitulate the structural and biophysical properties of the native complex (90, 93, 94). In the absence of high-resolution structures, the mechanism used by ClpP-ATPases to unfold and translocate substrates was deduced from those of related disaggregases, such as Hsp104 and ClpB (87, 88, 95, 96). Also, proteolytic machines with similar components such as the 26S and PAN proteasomes provided insights into the ClpP-ATPase interaction and potential mechanism of function (97, 98, 99, 100).

In this review, direct structural evidence for these mechanisms will be discussed using recently published cryoelectron microscopy structures of ClpP-ATPases from different species. We will summarize the structural elements that facilitate complex formation, substrate recognition, unfolding, and translocation. We will then evaluate mechanistic models that link ATP hydrolysis, IGF loop binding, substrate unfolding, and translocation and discuss their strengths and limitations. Finally, we will describe the intricate allostery of the tetradecameric ClpP complex as revealed by its behavior in the presence of small molecules and gain/loss-of-function mutations. Together, these structural data are expected to aid ongoing drug design efforts that target the ClpP-ATPase complex in bacterial infections and cancers.

## Part I. Structural basis for ClpP regulation by ATPase chaperones

### Current cryoEM structures of ClpP-ATPase complexes

Structures of ClpP-ATPase complexes from three bacterial species have been determined by cryoEM. The first to be published was the structure of ClpXP1P2 of *Listeria monocytogenes* (LmClpXP1P2), refined to an average resolution of 4.0 Å (101). The protein construct used to obtain this structure consists of an inactive S98A mutant of LmClpP1P2 heterocomplex, consisting of a heptameric LmClpP1 bound to heptameric LmClpP2, cross-linked using glutaraldehyde to the full-length, Walker B mutant of LmClpX. In this structure, all ClpX subunits are in the ATP loadable conformation bound to LmClpP2 heptamer, resulting in a flat LmClpX hexamer on top of the LmClpP1P2 tetradecamer. In addition, the ZBDs of two LmClpX hexamers oligomerize, causing two LmClpXP1P2 complex units to form head-to-head dimers of unknown biological relevance. The resolution of LmClpX (6–7 Å) is insufficient to determine the presence of bound nucleotides. The ZBDs in LmClpX were ultimately not modeled and, hence, not included in the PDB file, although low-resolution density for these domains was observed.

The second cryoEM structure is of ClpXP of *Neisseria meningitidis* (NmClpXP), refined to 2.3 to 3.3 Å resolution, using a full-length E185Q Walker B mutant of NmClpX and wild-type NmClpP, incubated with MgATP and SsrA-tagged green fluorescent protein (GFP) (102). Two distinct conformations of NmClpXP were resolved based on the position of the NmClpX subunit at the seam and were interpreted to represent two different steps in the functional cycle. The seam position refers to the breakage point of the spiral; one NmClpX subunit can occupy either the upper or lower seam position depending on its distance from the *cis*-ClpP ring. The nucleotide-binding states of the six NmClpX subunits were also defined. In contrast with the LmClpX structure, the NmClpX structure does not have visible electron density for ZBDs. A similar spiral topology was observed for ClpX in the structure of *Escherichia coli* ClpXP (EcClpXP, 3.2–4.3 Å resolution), with well-defined nucleotide-binding states and containing a short stretch of peptide in the substrate channel (103). To assemble this complex, a single-chain pseudohexamer of EcClpX E185Q Walker B mutant was used to form a complex with wild-type EcClpP in the presence of ATP/ATPγS. Other EcClpXP structures with bound peptide bearing the SsrA-sequence ALAA of the tagged GFP substrate (GFP-G_3_YG_9_SENYALAA, SsrA residues underlined), or another sequence of the peptide bound at a lower section of the EcClpX channel, were determined (104). Finally, three distinct conformations of EcClpAP with bound substrate were refined to 2.7 to 3.3 Å, using wild-type enzymes, ATP/ATPγS, and the RepA-GFP substrate containing the first 25 amino acid residues of RepA (105). All three conformations show a spiral topology for the D1 and D2 rings of EcClpA. In addition, three distinct binding states were observed for the same IGF loop near H sites that were interpreted to correspond to different steps in the functional cycle.

### Overall structure and the symmetry mismatch between ClpX and ClpP

**Figure 2:**
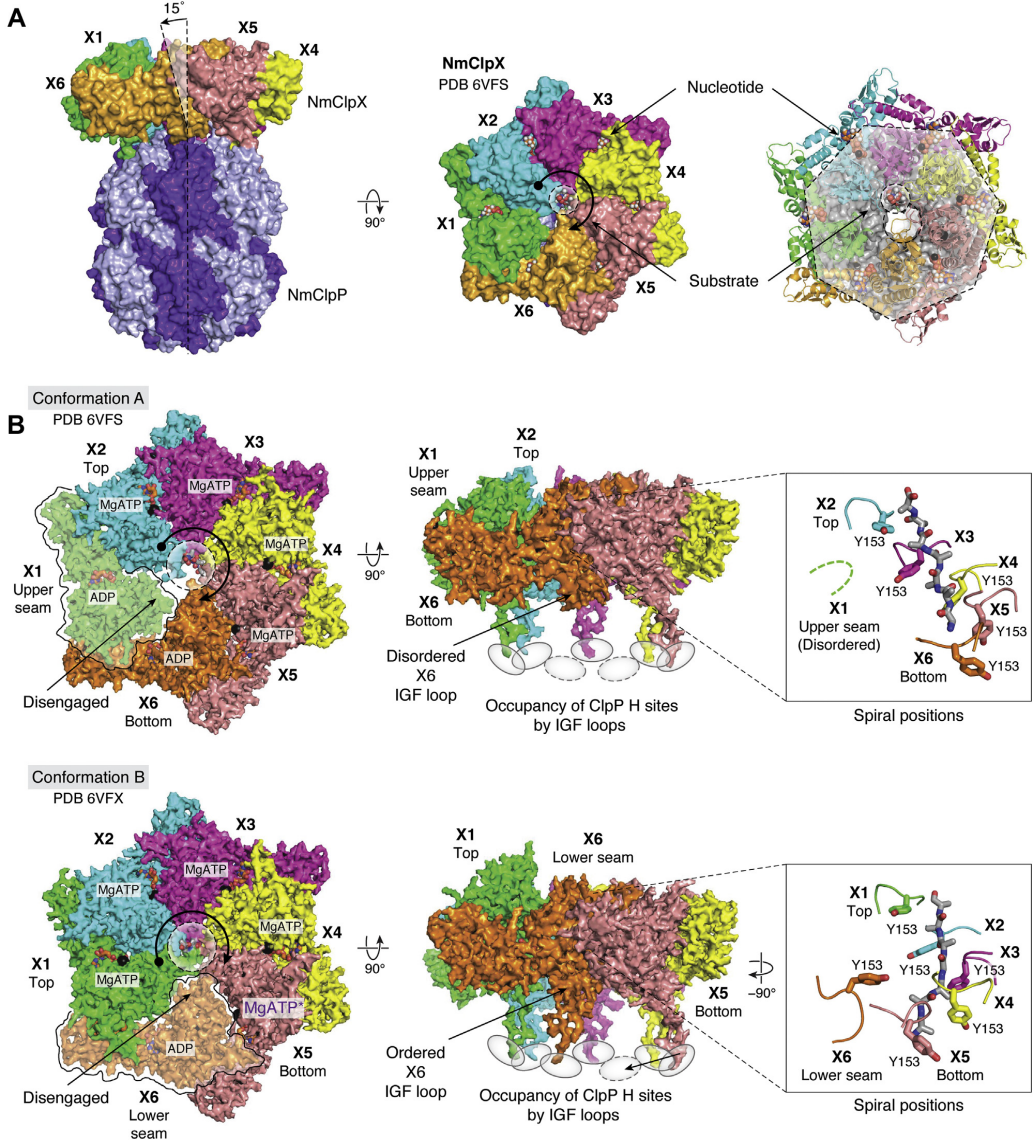
**General structure of a ClpP-ATPase complex.***A*, the cryoEM structure of *N. meningitidis* ClpXP (NmClpXP) shows hexameric NmClpX docked onto tetradecameric NmClpP. (*Left panel*) NmClpX (colored by subunit) forms a shallow *right-handed* spiral and docks on the apical surface of NmClpP (*coloredlight* and *dark purple* for contrast). Dashed lines indicate the pseudo-7-fold and 6-fold symmetry axes of NmClpP and NmClpX, respectively, that form a ∼15⁰ angle. (*Middle panel*) NmClpX binds six nucleotides (spheres) and a short peptide in the central channel (spheres). The nucleotide binding sites are formed by two adjacent NmClpX subunits. *Curved arrows* indicate the *right-handed* direction of the spiral when viewed along the pseudo-6-fold symmetry axis of NmClpX with NmClpP found below it. The round end of the *arrow* indicates the NmClpX subunit at the *top spiral* position (X2), and the *arrowhead* indicates the subunit at the bottom spiral position (X6). (*Right panel*) The offset between NmClpX and NmClpP is emphasized by drawing circles that delineate the rims (heptameric polygon) and entrance pore (*larger circle*) of NmClpP and the central channel of NmClpX (*smaller circle*). NmClpX is tilted and offset relative to the pseudo-7-fold symmetry axis of NmClpP. If ATP is bound, Mg^2+^ is also present (*black spheres*). The PDB ID for the structure is 6VFS. *B*, two conformations of NmClpXP were resolved by cryoEM based on the identity of the NmClpX subunit occupying the seam position, where the shallow spiral breaks, causing the subunit to disengage from the substrate (not touched by *curved arrows*). The images on the *left* are *topviews* of the complexes viewed along the pseudo-6-fold symmetry axis of NmClpX, with NmClpP at the *bottom* not shown for clarity. NmClpX subunits that occupy seam positions are indicated by a *bold contour line* and rendered opaque compared with other subunits. (*Left*) In Conformation A, NmClpX subunit X1 occupies the *upper* seam position. In Conformation *B*, NmClpX subunit X6 occupies the lower seam position. The *upper and lower* seam positions are distinguished based on the distance of the subunit from the ClpP apical surface, *i.e.*, at the lower seam position, the NmClpX subunit is closer to ClpP, hence its pore-1 and IGF loops are closer to the ClpP apical surface. At seam positions, the NmClpX subunit is ADP-bound and does not engage substrate. The two conformations also differ in the NmClpX subunit that occupies the top and *bottomspiral* positions (see *round end* and head of *curved arrows*). In Conformation A, four ATP and 2 ADP molecules are bound. In Conformation B, five ATP and one ADP molecules are bound. NmClpX subunit X5 in Conformation B binds ATP in a post-hydrolysis, pre-ADP release state (MgATP∗). *Broken circles* delineate the substrate channel. (*Middle*) The IGF loops of NmClpX are flexible and can dock/undock at ClpP H sites (*gray ovals*). In Conformation A, five IGF loops contact H sites, leaving two unoccupied sites (*broken ovals*). In Conformation B, six IGF loops contact H sites, leaving one unoccupied site. In Conformation A, the IGF loop of subunit X6 is disordered and does not contact an H site, but does so in Conformation B, suggesting a step-like movement. (*Right*) Pore-1 loops directly engage the substrate in a *right-handed* spiral arrangement going from top to *bottom spiral* position, intercalating the substrate every two residues using residue Y153. In Conformation A, the pore-1 loop of the disengaged, upper seam subunit X1 is disordered and not modeled in the structure (*broken green lines*). In Conformation B, the pore-1 loop of the *upper seam* subunit X6 is disengaged from substrate (*orange loop*) but has visible electron density. The PDB IDs for the structures are 6VFS (NmClpXP Conformation A) and 6VFX (NmClpXP Conformation B). CryoEM, cryoelectron microscopy; NmClpP, *Neisseria meningitidis* ClpP protease.

Both conformations of NmClpXP show one NmClpX hexamer bound to an NmClpP tetradecamer (102) (Fig. 2, *A* and *B*). Although full-length NmClpX was used during sample preparation, the ZBDs are not visible in the structures due to domain flexibility. In both structures, NmClpX is tilted and laterally shifted relative to NmClpP, causing the bound peptide to approach the substrate channel at an angle of ∼15 degrees relative to the long axis of ClpP (Fig. 2*A*). Misalignment of asymmetric NmClpX and NmClpP rings causes the substrate translocation channel to twist and constrict at the interface, as is also observed in the structures of LmClpXP1P2 and PAN proteasome (99, 101). Unlike the crystal structure of EcClpX pseudohexamer that shows a twofold symmetric dimer of trimers, the cryoEM structure NmClpX shows a shallow, right-handed spiral with pseudo-6-fold symmetry (86). This arrangement causes conserved pore-1 loops lining the substrate channel to form a spiral around the bound peptide (Fig. 2*B*). It is thought that when the substrate polypeptide reaches ClpP, ClpX and ClpP will align.

The main difference between the two conformations is in the NmClpX subunit that occupies the seam position of the spiral and in the distance of that subunit from ClpP (Fig. 2*B*). This is easily visualized by looking only at the pore-1 loops of each NmClpX subunit, which harbors the conserved Y153 residue that interacts directly with substrate (Fig. 2*B*, right). In Conformation A, subunit X1 occupies the upper seam position and is disengaged from substrate, while subunits X2 and X6 occupy the top and bottom spiral positions, respectively (Fig. 2*B*). In Conformation B, subunit X6 occupies the lower seam position and is disengaged from substrate, while subunits X1 and X5 occupy the top and bottom spiral positions, respectively. Thus, there are at least two seam positions, which would suggest an upward clockwise movement of a seam subunit to the top position.

**Figure 3:**
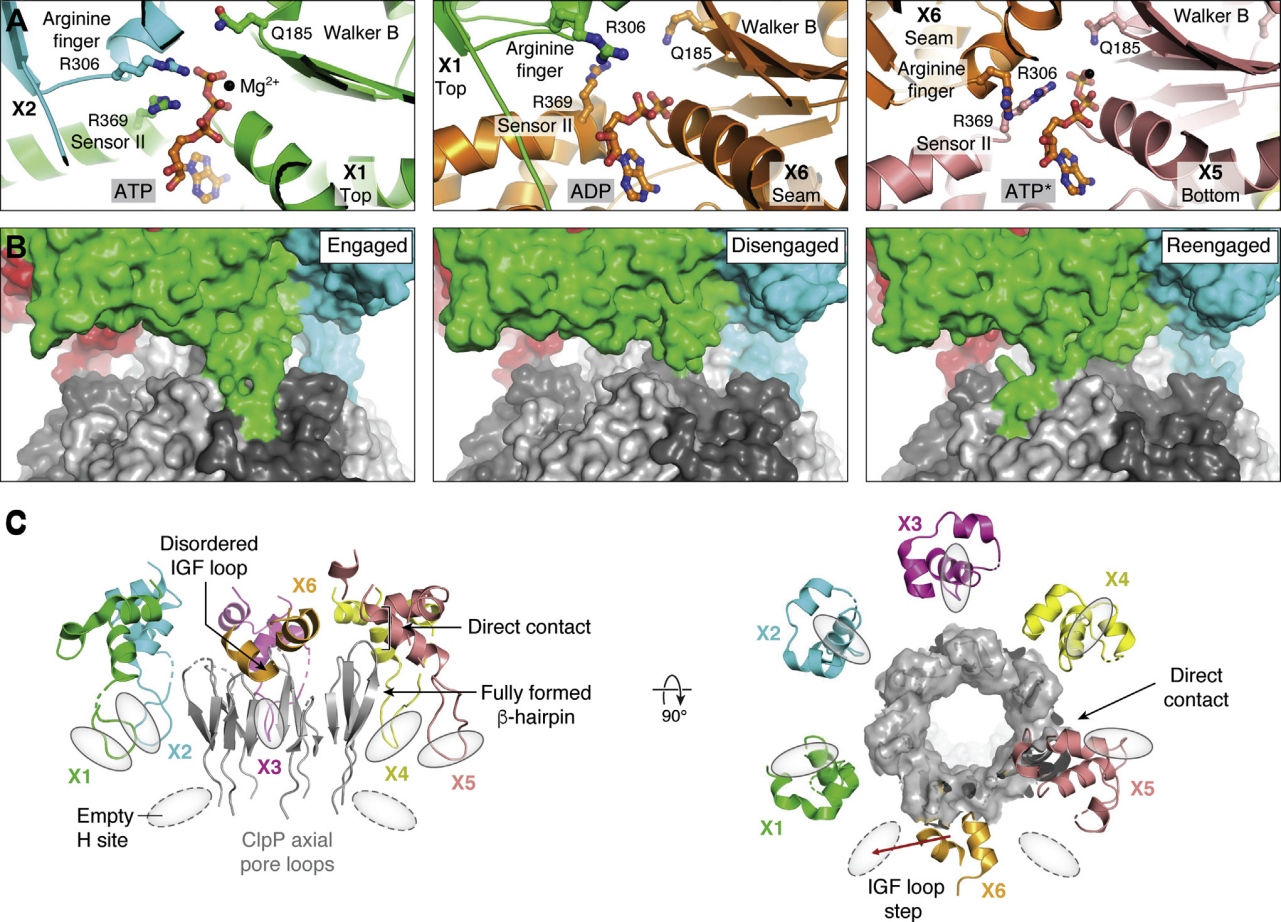
**Nucleotide binding and IGF loop interactions with H sites.***A*, different nucleotide-ATPase interactions occur depending on the nature of the bound nucleotide. The structure of NmClpX Conformation B exemplifies these interactions (PDB ID 6VFX). (*Left*) In an ATP hydrolysis-active site, the nucleotide is stabilized by the Sensor II R369 of subunit X1 and the arginine finger R306 from the clockwise subunit X2. The arginine residues bind the γ-phosphate of ATP. Additional stabilizing contacts by the Walker A (not shown) and Walker B motifs are required to properly position the nucleotide for hydrolysis. The Walker B residue E185 is essential for ATP hydrolysis and is mutated in NmClpX to Q185 to aid complex assembly by impairing hydrolysis. The bound Mg^2+^ ion is shown as a *black sphere*. (*Middle*) After ATP hydrolysis, nucleotide-stabilizing interactions and Mg^2+^ are removed to facilitate ADP release. The binding site shown here is formed by subunits X6 (*lower seam position*) and X1 (*top position*). (*Right*) In subunit X5, located at the *bottom spiral* position (the position where ATP hydrolysis is proposed to always occur under the SC/2R mechanism framework, see Fig. 5), the electron density of the bound nucleotide is less resolved than those for other bound nucleotides. Due to the movement of subunit X6 to the seam position, the nucleotide binding site is distorted. The bound nucleotide here is interpreted as an unreleased, post-hydrolytic ATP species (MgATP∗ or MgADP+Pi). The PDB ID for the structure used in this image is 6VFX. *B*, three conformations of EcClpAP were resolved and describe the step-like movement of an IGF loop after ATP hydrolysis in the same subunit. The IGF loop of one EcClpA subunit is engaged with an EcClpP H site in the first conformation, disengaged in the second, and re-engaged with the next clockwise H site. The PDB IDs for the structures are 6W22 (engaged), 6W23 (disengaged), and 6W24 (reengaged). *C*, the IGF loops of NmClpX mediate flexible interactions with the apical surface of NmClpP. (Left of arrow) In NmClpX Conformation A, the IGF loops of five NmClpX subunits (X1-X5, *colored* by chain) contact H sites on NmClpP (*gray ovals*) and cause the ordering of the nearby NmClpP N-terminal loops (*gray* β-hairpins). The IGF loop of subunit X6 is disordered and not modeled, while that for subunit X5 directly contacts a nearby β-hairpin, causing the latter to become fully ordered. The IGF loop of subunit X6 is putatively located between two empty H sites, suggesting a clockwise stepping motion to occupy the leftmost, empty H site. This stepping motion moves subunit X6 to the lower seam position and subunit X5 to the *bottom position*, in effect, similar to Conformation B (see Fig. 2*B*). (Right of arrow) The same structure as the one on the left, but with the β-hairpin structures of NmClpP shown as surfaces to emphasize the offset between NmClpX and NmClpP and the contact between the IGF loop of subunit X5 and the surface of an axial pore β-hairpin. The flexibility of IGF loops enables the tilting and offset between NmClpX and NmClpP rings and is important for a functional complex. The PDB ID for the structure is 6VFS. EcClpP, *Escherichia coli* ClpP protease; IGF, isoleucine–glycine–phenylalanine; NmClpP, *Neisseria meningitidis* ClpP protease.

The nucleotides bound to NmClpX subunits are well defined in the structures (Figs. 2*B* and 3*A*) (102). Nucleotide assignment is based not only on electron density but also on known interactions between nucleotides and conserved ATPase domains (Figs. 1*B* and 3*A*) (86). For instance, in NmClpX Conformation B, the Sensor II arginine (R369, subunit X1) and the Sensor-I arginine finger (R306) from an adjacent clockwise subunit (X2) interact with the β- and γ-phosphates of ATP (Fig. 3*A*, left). When ADP is bound, both arginine residues move away from nucleotide (Fig. 3*A*, middle). A ClpX subunit is in the ATP loadable conformation and is, therefore, hydrolytically active only when the Sensor II arginine, Arginine finger, and the Walker A and B motifs are properly oriented (Figs. 1*B* and 3*A*). In Conformation A, subunits X2, X3, X4, and X5 are ATP-bound, while subunit X1 at the upper seam position and subunit X6 at the bottom position are ADP-bound (Fig. 2*B*). In Conformation B, subunits X1, X2, X3, and X4 are ATP-bound, while subunit X6 at the lower seam position is ADP-bound (Fig. 2*B*). Interestingly, subunit X5 at the bottom position is interpreted to bind ATP in a posthydrolysis state (ATP∗ or ADP+Pi) due to the nucleotide’s less defined density that is larger than that for an ADP species (Figs. 2*B* and 3*A*, right). In general, in NmClpXP structures, NmClpX subunits at seam positions bind ADP, while those at the bottom position bind either ADP or ATP∗. NmClpX subunits in other spiral positions are all ATP-bound (Fig. 2*B*).

The flexibility of IGF loops allows NmClpX to maintain the dynamic spiral arrangement while remaining multivalently bound to NmClpP (Fig. 2*B*) (101, 102). The IGF loops can extend and retract as the subunits change positions within the spiral. For one particular IGF loop in the EcClpA subunit, engagement with and disengagement from a single EcClpP H site, followed by re-engagement with an adjacent, clockwise H site, were observed in three different conformers of the EcClpAP complex (Fig. 3*B*, green ClpA subunit). The other five IGF loops remain engaged to an H site in the three conformers (Fig. 3*B*, other colored ClpA subunits) (105). Similar movements were observed in NmClpXP structures. In Conformation A of NmClpXP, the IGF loop of subunit X6 is disengaged, leaving two open H site positions (Fig. 2*B*). In Conformation B, the IGF loop of subunit X6 now occupies one of the two previously empty H sites in a clockwise step (Fig. 2*B*). Thus, all six IGF loops engage six out of seven H sites. Like springs, the IGF loops facilitate the shifting between positions within the NmClpX spiral and the offsetting of NmClpX and NmClpP rings (Fig. 2*A*). IGF loop flexibility is therefore key in the formation of a functional complex despite the symmetry mismatch between NmClpX and NmClpP rings (101, 102, 103, 105). It is not clear why such asymmetry between components persists in proteolytic machines.

Interestingly, a specific role for the nonconserved, long C-terminus of LmClpP2 was discovered in the structure of LmClpXP1P2 (101). LmClpX IGF loop binding to an LmClpP2 H site causes the C-terminus of the same LmClpP2 subunit to form an extended structure. In the absence of IGF loop binding, the C-terminus forms a compact structure that can occupy the H site and protect its hydrophobic environment from solvent. Truncation of the C-terminus reduces LmClpP2 solubility, while shorter deletions increase its affinity for LmClpX. ClpPs from other organisms with naturally shorter C-termini have higher affinities for ClpX (101). Thus, the longer C-terminus of LmClpP2 appears to be a specific feature that modulates its binding affinity to LmClpX and might be targeted for antibiotic specificity.

### ClpP pore opening induced by ClpX binding

In the apo state, the N-terminal loops, also called axial pore loops, of ClpP block its axial pores and gate substrate entry (83). In many crystal structures, the binding of small-molecule agonists to H sites (*e.g.*, ADEPs) causes ordering of N-terminal loops into β-hairpin turns, resulting in an open gate conformation (83). This allosteric effect is also observed in the structure of NmClpXP, where six β-hairpin turns are formed, with the one in direct contact with the IGF loop of NmClpX subunit X5 being the most ordered due to stabilizing interactions (Fig. 3*C*) (102). In EcClpAP complexes with a single EcClpA hexamer, the EcClpP ring *cis* to EcClpA is in the open gate conformation (∼25 Å diameter), while the *trans* ring is in the closed gate conformation (∼15 Å diameter). Doubly capped EcClpAP complexes have open pores in both ClpP rings (105). Hydrogen-deuterium uptake of LmClpX IGF loops is reduced upon complex formation with LmClpP, suggesting solvent-shielding interactions with axial pore loops as also seen in NmClpXP structures (Fig. 3*C*) (101).

### SsrA degron recognition by EcClpXP

In *E. coli* and other eubacteria, the C-terminal SsrA-tag (AANDENYALAA) is added by the tmRNA to prematurely arrested proteins for ClpXP degradation (35, 36). During ribosome stalling, tmRNA binds to the ribosomal A site and catalyzes a transpeptidation reaction that adds an alanine residue to the nascent polypeptide. The tmRNA then displaces the original mRNA with an open reading frame containing the coding sequence for the rest of the SsrA tag and a stop codon for the recruitment of translation termination factors. The SsrA-tagged protein is then released from the ribosome and degraded mainly by ClpXP.

**Figure 4:**
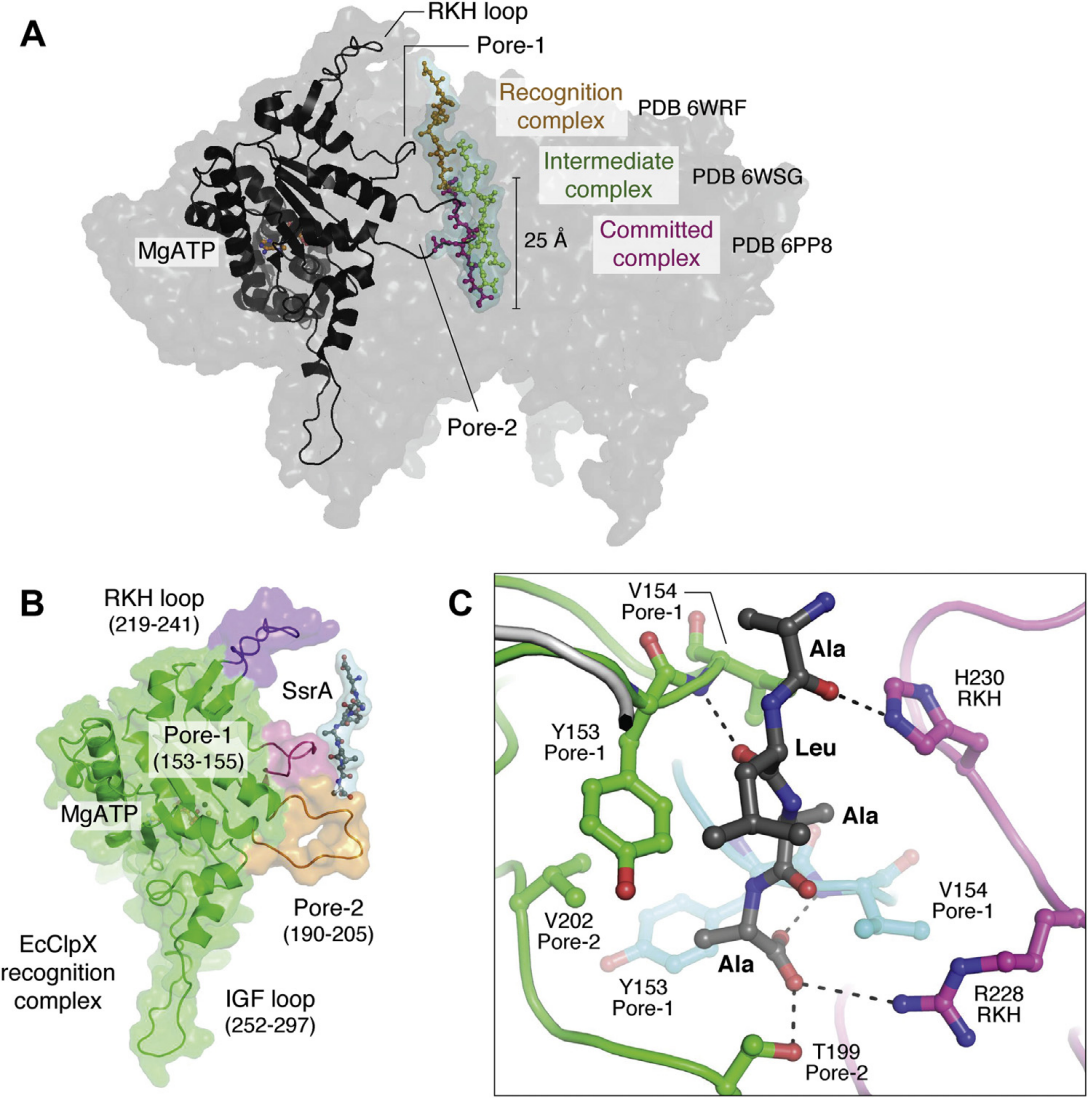
**Intermediates in substrate recognition and translocation by EcClpX.***A*, three cryoEM structures corresponding to different steps in the degradation of SsrA-tagged protein substrates have been determined (103, 104). The first structure corresponds to a recognition step where the peptide (*gold sticks*) is bound at the top of the channel. The second structure corresponds to an intermediate step where the substrate (*green sticks*) is translocated by ∼25 Å relative to its position in the recognition complex. The third structure corresponds to a committed step during which the unfolded substrate (*magentasticks*) is preparing for entry into the ClpP chamber for degradation. For simplicity, the surface representation shown is that for the EcClpX recognition complex only, with one subunit displayed in cartoon representation. The relative positions of the bound peptide substrates in the intermediate and committed complexes were obtained by superposition of the three complex structures. The PDB IDs for the structures are 6WRF (recognition complex), 6WSG (intermediate complex), and 6PP8 (committed complex). *B*, in the EcClpX recognition complex, the RKH, pore-1, and pore-2 loops of the subunit occupying the top spiral position directly engages the substrate. The pore-2 loop prevents further translocation by blocking the channel, allowing EcClpX to scan the bound protein for the SsrA degron. The recognition step is reversible so that captured proteins without the SsrA sequence can be released. *C*, specific residues unique to EcClpX and located in the RKH, pore-1, and pore-2 loops directly participate in SsrA sequence recognition. Shown here are the last four residues of the SsrA degron (Ala-Leu-Ala-Ala, *gray sticks*). Both van der Waals and hydrogen bonding interactions are used by the loops to identify the SsrA degron. Because of the specificity of this interaction, mutations in either the SsrA sequence or the highlighted residues in the loops (shown as *colored sticks*), result in a decrease in binding affinity between EcClpX and substrate. ClpP, caseinolytic protease P; RKH, arginine–lysine–histidine.

A fluorescence quenching assay using labeled EcClpXP and SsrA-tagged substrates showed the existence of at least three distinct steps during degradation (Fig. 4*A*) (106). The first corresponds to an ATP-independent recognition step in which EcClpX scans the protein for the SsrA degron (Fig. 4, *B* and *C*). This step is reversible, allowing binding and release of non-SsrA tagged proteins. Earlier biochemical studies showed that the last two Ala residues of the SsrA degron are most important for recognition and degradation by EcClpXP (107). Similar degrons ending in Ala-Ala-COO^-^ efficiently mark proteins for EcClpXP degradation, where specific residues in the RKH, pore-1, and pore-2 loops are involved in recognition (Fig. 4, *B* and *C*) (108, 109, 110). Subsequent steps correspond to ATP-dependent processes in which the substrate is pulled toward the EcClpX opening, then unfolded and translocated into the substrate channel. Two of these steps are interpreted to correspond to intermediate and committed complexes (Fig. 4*A*) (106).

The structure of a recognition complex provides the basis for SsrA sequence identification by EcClpXP (104). The SsrA degron is bound at the top of the EcClpX substrate channel, gripped by the RKH, pore-1, and pore-2 loops (Figs. 1*B* and 4, *B* and *C*). Of particular interest is the pore-2 loop of the topmost, ATP-bound subunit that extends toward the channel axis and blocks further substrate translocation (Fig. 4*B*). In the structure of the intermediate complex, this blocking pore-2 loop is moved out of the way of the translocating substrate, and the corresponding subunit is no longer nucleotide bound. The substrate is also moved ∼25 Å down the channel, indicative of an ATP hydrolysis-generated power stroke that drives translocation (Fig. 4*A*) (104). Other structures of EcClpXP and NmClpXP show the bound peptides further down the substrate channel and are interpreted as committed complexes in which the unfolded substrates prepare for entry into the ClpP chamber (Fig. 4*A*) (102, 103).

In the recognition complex, residues T199 and V202 of the blocking pore-2 loop in EcClpX form van der Waals and hydrophobic interactions with the ultimate Ala side chain in the SsrA degron. The hydroxyl group of the same T199 residue and the peptide bond amino group of V154, in the pore-1 loop of a nearby subunit, form hydrogen bonds with the terminal carboxylate group of the SsrA degron. Other interactions with the SsrA degron involve side or main chains of Y153 and V154 from nearby pore-1 loops and R228 and His230 of nearby RKH loops (Fig. 4*C*) (102).

Mutations that abolish the above interactions have a significant effect on the affinity of EcClpX for SsrA-tagged substrates. In modifying the SsrA sequence, the K_M_ value for a 29-residue substrate is dramatically increased upon mutation of the ultimate and penultimate Ala residues, with little effect on V_max_ (102). Mutation of the upstream Leu and Tyr residues of SsrA has a modest effect on K_M_ (107). In mutating EcClpX residues that directly contact the SsrA degron (Y153A, V154F, R228A), the K_M_ values increase by at least 50-fold (111, 112, 113, 114). Additional mutations based on interactions first observed in the recognition complex (T199A, T199V, V202A, H230A) cause large increases in K_M_ for the GFP-SsrA substrate, while the conservative T199S mutation does not (∼4-fold) (104). The R228A mutation in the RKH loop reduces EcClpX specificity for SsrA-tagged substrate but increases that for a substrate containing the λO N-terminal degron (112). Notably, human ClpX lacks activity toward SsrA-tagged substrates, likely due to Leu mutations at sites corresponding to the T199 and H230 residues of EcClpX (Fig. 4*C*). Substitution of human RKH and pore-2 loops with corresponding sequences from EcClpX confers the SsrA-recognition activity on HsClpX (113).

### Mechanisms for substrate translocation

**Figure 5:**
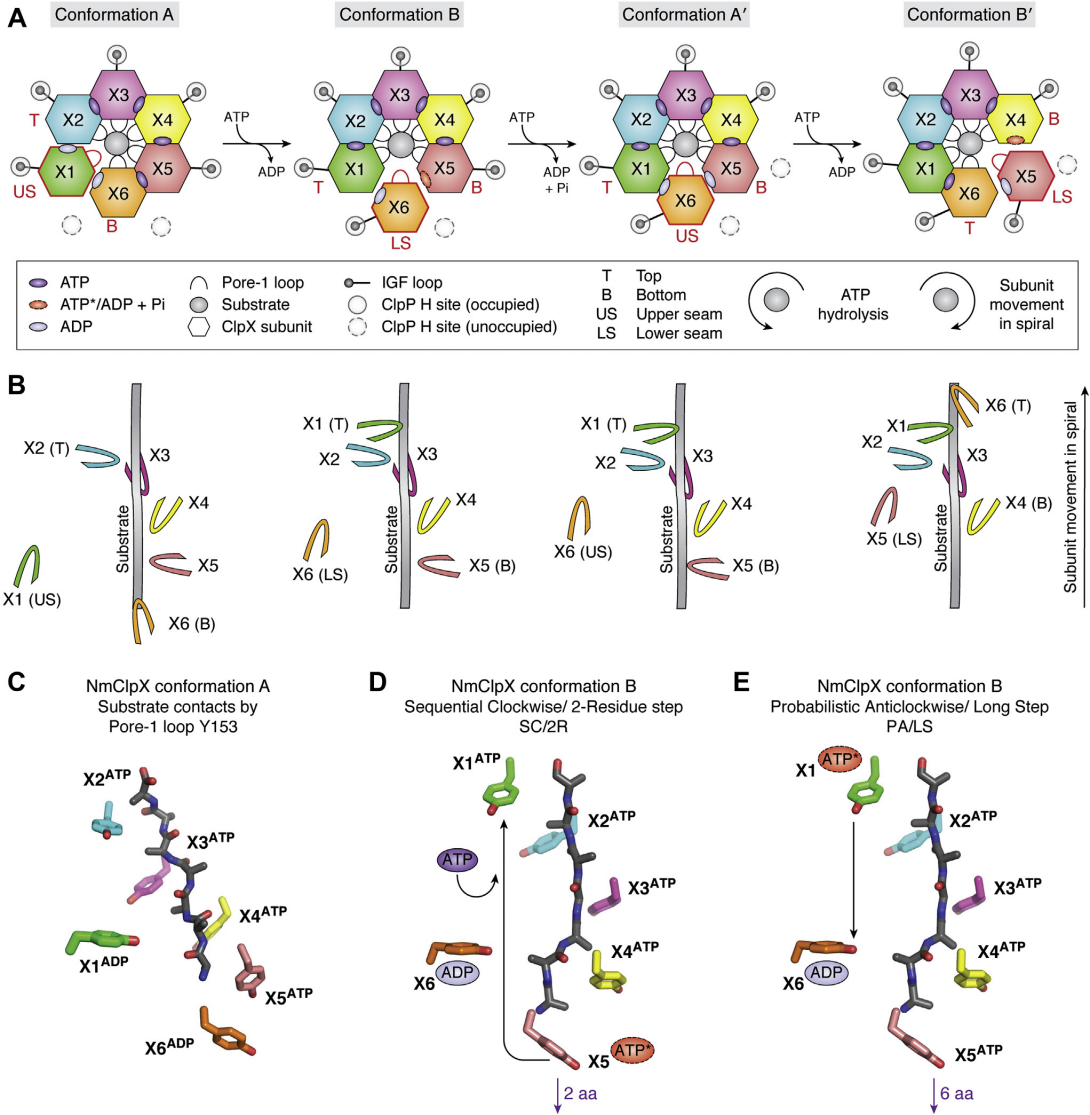
**Possible mechanisms for substrate translocation used by ClpP-ATPase complexes.***A*, substrate translocation catalyzed by NmClpX occurs with the clockwise movement of subunits through spiral positions, arising from sequential ATP hydrolysis proceeding in the opposite direction. Hydrolysis always occurs at the bottom (denoted as B) spiral position and enables the stepping of an IGF loop from one H site to an adjacent, clockwise H site. The two conformations of NmClpX captured by cryoEM represent two intermediates in this process. In Conformation A, subunit X6 is at the *bottom* (B) *spiral* position, subunit X2 is at the *top* (T) *spiral* position, and subunit X1 occupies the *upper seam* (US) position. Transition to Conformation B involves exchange of ADP (*light purple ovals*) for ATP (*dark purple ovals*) in subunit X1, moving it from the US to the T spiral position, and subunit X6 from the B to the lower seam (LS) position. An ATP hydrolysis event (*broken red oval*) will occur next at the B spiral position now occupied by subunit X5 in Conformation B. After ATP hydrolysis in subunit X5 (denoted by the presence of bound ADP, *light purple oval*), the clockwise subunit X6 moves to the US position, while subunit X1 is still at the top position (Conformation A′). Transition from Conformation A to Conformation B then back to Conformation A (or A′) uses one ATP and translocates two amino acid residues in the substrate channel. The stepping of an IGF loop from one H site (*gray circles*) to another occurs clockwise as ATP is hydrolyzed in the NmClpX subunit at the B spiral position. In Conformation A of NmClpX, ATP has been previously hydrolyzed at the B spiral position (subunit X6, ADP-bound), and the IGF loop has retracted from an H site and is preparing to occupy the next clockwise H site, leaving two empty H sites (*broken gray circles*). In Conformation B, the same IGF loop has occupied an H site, leaving only one empty H site. The process repeats as NmClpX returns to Conformation A′. *B*, the pore-1 loops of NmClpX directly engage the substrate. In Conformation A of NmClpX (PDB ID 6VFS), five pore-1 loops (of X2, X3, X4, X5, X6) form a clockwise spiral around the substrate, with each loop intercalating the substrate every two residues. The pore-1 loop of subunit X2 is at the top of the spiral, while that of subunit X6 is at the bottom. Subunit X1 occupies the *US* position and its pore-1 loop is disengaged from substrate. The pore-1 loop of subunit X1 is not modeled in the structure due to weak density but is shown as a cartoon in the image. In Conformation B (PDB ID 6VFX) of NmClpX, the pore-1 loops have re-arranged due to subunit movements in the spiral. The pore-1 loop of subunit X1 is at the top of the spiral, while that of subunit X5 is at the bottom. The pore-1 loop of subunit X6 is disengaged from the substrate, but its density is sufficiently clear to model the loop at this position. The pore-1 loops rearrange according to the subunit movements in the spiral shown in (*A*). *C*, residue Y153 of NmClpX spirals around the bound substrate and intercalates it every two amino acid residues. This figure is in a similar orientation to the leftmost image in *B* and shows the putative position (shadowed image) of the Y153 residue of subunit X1 in the spiral. The pore-1 loop of X1 has weak electron density in NmClpX Conformation A. *D*, in the sequential clockwise/2-residue step mechanism (SC/2R), hydrolysis at the *B position* (subunit X5 in NmClpX Conformation B) moves the clockwise seam subunit X6 towards the *USposition* (net result is the same as Conformation A′, with two bound ADPs). ADP release and ATP-rebinding at the *USposition* moves the same subunit to the *Tposition* (Conformation B′). Each transition from Conformation A to B to A′ results in a 2-residue translocation step for the substrate. *E*, In the probabilistic anticlockwise/long step (PA/LS) mechanism, ATP hydrolysis can occur at any position in the NmClpX spiral. Shown here using the same structure as in D is a hypothetical ATP hydrolysis event that occurs at the *Tposition*, occupied by subunit X1, that pushes the same subunit down a 6 to 8 residue distance in the substrate channel to occupy the seam, then the bottom spiral position. The net result is an anticlockwise movement of NmClpX subunits. Random, simultaneous ATP hydrolysis events at different spiral positions can generate power strokes enough to translocate as long as 24 residues in a single step.

The structures of NmClpXP show bound substrates in the NmClpX channel gripped by spiraling pore-1 loops and also show IGF loops that appear to indicate a sequential stepping motion on NmClpP H sites (Figs. 2*B* and 5, *A*–*C*). The pore-1 loop residue Y153 directly interacts with substrate polypeptide at every two amino acid residues when engaged (Figs. 2*B* and 5*C*). In Conformation A, the pore-1 loops of subunits X2, X3, X4, X5, and X6 engage the substrate, with the pore-1 loop of subunit X2 located at the top of the spiral, and the pore-1 loop of subunit X6 at the bottom, closest to NmClpP (Fig. 5, *A* and *B*). The pore-1 loop of subunit X1 (upper seam position) is disengaged from substrate (Fig. 5, *A* and *B*). For a pore-1 loop to disengage from substrate, it must form a short α-helix and retract from the central channel. In Conformation B, the pore-1 loop of subunit X6 (lower seam position) is disengaged from the substrate, while those of subunits X1, X2, X3, X4, and X5 are engaged (Fig. 5, *A* and *B*). The pore-1 loop of subunit X1 is at the top of the spiral, while the pore-1 loop of subunit X5 is at the bottom (Fig. 5, *A* and *B*). Additional interactions with substate are formed by the pore-2 loop of subunit X1 and the RKH loop of subunit X5, but not by the pore-2 or RKH loops of other subunits (not shown). In contrast, the structures of EcClpXP show more pore-2 and RKH loops engaging the substrate together with pore-1 loops. This is interpreted to arise from the specificity of EcClpX for the SsrA degron (Fig. 4, *B* and *C*) (103, 104).

The structures of NmClpXP suggest a rotary model for substrate translocation that is consistent with those of EcClpXP, EcClpAP, and related disaggregases (103, 105, 115). This *sequential clockwise/2-residue step* (SC/2R) mechanism can be understood by looking at Conformations A and B in Figure 5*A*. In Conformation A, subunit X6 at the bottom spiral position is ADP-bound and engages the substrate. Its IGF loop does not contact an H site but is poised to occupy one of the two empty ones (left broken circle). Its clockwise neighbor, subunit X1, occupies the upper seam position, is ADP-bound, and does not engage the substrate (Fig. 5, *A* and *B*). ClpX subunits at seam positions (upper and lower) are always ADP-bound and disengaged from substrate.

In Conformation B, subunit X6 has moved clockwise (from the bottom spiral position in Conformation A) to the lower seam position, with its IGF loop now bound to an H site (Fig. 5*A*). Its clockwise neighbor, subunit X1, has moved upward from the upper seam position to the top spiral position, binds ATP, and now engages the substrate with its pore-1 loop (Fig. 5, *A* and *B*). This transition from Conformation A to Conformation B is thus mediated through nucleotide exchange in the upper seam position. The bottom spiral position in Conformation B is occupied by subunit X5 that contains ATP∗ (posthydrolysis state). In the cryoEM structure, the IGF loop of subunit X5 is more extended than those of other subunits, indicating tension to be relieved by a forward, clockwise step to an adjacent H site (Fig. 5*A*). Upon ATP hydrolysis in subunit X5, ADP is formed and the IGF loop retracts from the H site (Conformation A′), an equivalent state to that of subunit X6 in Conformation A (Fig. 5, *A* and *C*). Subsequently, the clockwise neighboring subunit (X6) moves to the upper seam position (equivalent position to subunit X1 in Conformation A) (Fig. 5*A*). A transition from Conformation A to Conformation B to Conformation A′ has therefore occurred with one ATP hydrolyzed (Fig. 5*A*) (102). A new nucleotide exchange event at the upper seam subunit X6 of Conformation A′ moves this subunit to the top position, while the IGF loop of subunit X5 takes a step to a clockwise H site and moves to the lower seam position from the bottom spiral position (Conformation B′). A new ATP hydrolysis event will occur at the new bottom spiral position now occupied by subunit X4 (Fig. 5*A*).

Thus, in the proposed SC/2R mechanism, ATP hydrolysis always occurs in the ClpX subunit at the bottom spiral position, which allows its IGF loop to step one H site clockwise (Fig. 5, *A*, *B*, and *D*). This one step moves the bottom subunit to the lower seam position, and the next, anticlockwise subunit to the bottom spiral position where ATP hydrolysis will occur next (Fig. 5*A*). Two substrate residues are pulled down the channel with each subunit exchange at the bottom spiral position (Fig. 5*D*). Nucleotide exchange (ADP for ATP) moves the subunit at the upper seam position to the top spiral position. As one ATP is hydrolyzed per IGF loop step, seven ATPs must be hydrolyzed for each ClpX subunit to pass through the bottom spiral position and reset the process. This sequential, clockwise movement of ClpX subunits through spiral positions caused by the anticlockwise ATP hydrolytic cycles leads to the processive hand-over-hand mechanism for substrate translocation (Fig. 5, *A*–*D*) (115).

### Alternative mechanisms for substrate translocation

The SC/2R mechanism for substrate translocation is the consensus mechanism for AAA+ motors with spiral topologies and substrate contacts now seen in ClpP-ATPase complexes. However, this mechanism seems to be in discord with the longer, 6-residue translocation steps measured in optical trapping experiments for EcClpXP. Even longer, 24-residue steps, interpreted as kinetic bursts, have been recorded (103, 116, 117, 118, 119, 120, 121, 122). Considering the measured steady-state rate for ATP hydrolysis of 3.6 s^−1^ (rate constant of 0.28 s) under the same conditions, a 6-residue basic translocation step would take at least 0.8 s within the framework of the SC/2R mechanism. This disagrees with the short translocation time of 0.1 s measured for EcClpXP in optical traps using a multidomain filamin-A substrate (116). In other words, the SC/2R mechanism predicts a slower translocation motor than would be required by the much faster translocation rates observed experimentally. Moreover, EcClpX pseudohexamers with only two catalytically active subunits do not stall, in disagreement with the SC/2R mechanism, which presupposes that ATP hydrolysis occurs at only one position in the spiral at any time during translocation (102, 123).

To resolve this conflict, a *probabilistic anticlockwise/long step (PA/LS)* mechanism was proposed based on the structure of EcClpXP that is similar to Conformation B of NmClpXP. In the PA/LS model, a hypothetical scenario such as the one depicted in Figure 5*E* can occur, in which ATP hydrolysis at the top position results in a power stroke that causes it to move anticlockwise to the seam position, leading to a translocation step of 6 to 8 residues (103). Random ATP hydrolysis at other positions may generate strain in the spiral that can force the top subunit to perform the power stroke to relieve strain, and multiple such events can explain fast kinetic bursts. In this, the PA/LS model eliminates the requirement for sequential action, although it can still occur under this framework.

The hypothetical example above requires that other subunits must lose their grip on the substrate in a zipper-like motion to enable the top subunit to grip and push the substrate down the channel (103). It is not clear how ATP hydrolysis could trigger this, and no structures for these predicted intermediates exist. Furthermore, both anticlockwise and stochastic movements of ClpX would result in intermediates where empty H sites and seam subunits would be offset. However, in all ClpP-ATPase structures, the ATPase subunit at the seam position is always aligned close to an empty H site, in disagreement with the PA/LS model (Figs. 2*B* and 5*A*).

On the other hand, current ClpP-ATPase structures used to support the SC/2R mechanism show narrow substrate channels unable to fit certain types of substrates for which 2-residue translocation steps may not necessarily apply. For instance, disulfide-bonded, knotted, and polyproline tract containing substrates can be translocated and degraded by ClpXP (124, 125, 126, 127, 128). These substrates require wider channels that likely result in the distortion of the ATPase spiral. Furthermore, cross-linking EcClpP to EcClpA was found to still support substrate unfolding, translocation, and degradation albeit at lower rates compared with uncross-linked complexes (129). This indicates that rotation of EcClpA relative to EcClpP is not required for these functions, as would be expected from genetically fused AAA+ proteases such as Lon (130, 131). It has been proposed that a spring-like mechanism mediated by IGF loops at the ClpP-ATPase interface might be sufficient to support functions without rotation between enzyme components (103).

Clearly, more intermediate structures for ClpP-ATPases need to be determined as neither proposed mechanism seems to reconcile all available structural and biochemical data. The specific event that triggers ATP hydrolysis in either mechanism is also unknown (132). It seems possible that ClpP-ATPases might operate through different mechanisms under different experimental conditions (102, 103).

## Part 2. Allosteric regulation and conformational selection of ClpP by small molecules and gain/loss-of-function mutations

As discussed in the previous section, spurious protein degradation by ClpP is prevented by the direct screening of substrates by the partner ATPase and by ensuring that ClpP is only active upon formation of the complex with it. ClpP has a highly allosteric chamber that confines the catalytic sites, which adds another layer to its regulation.

**Figure 6:**
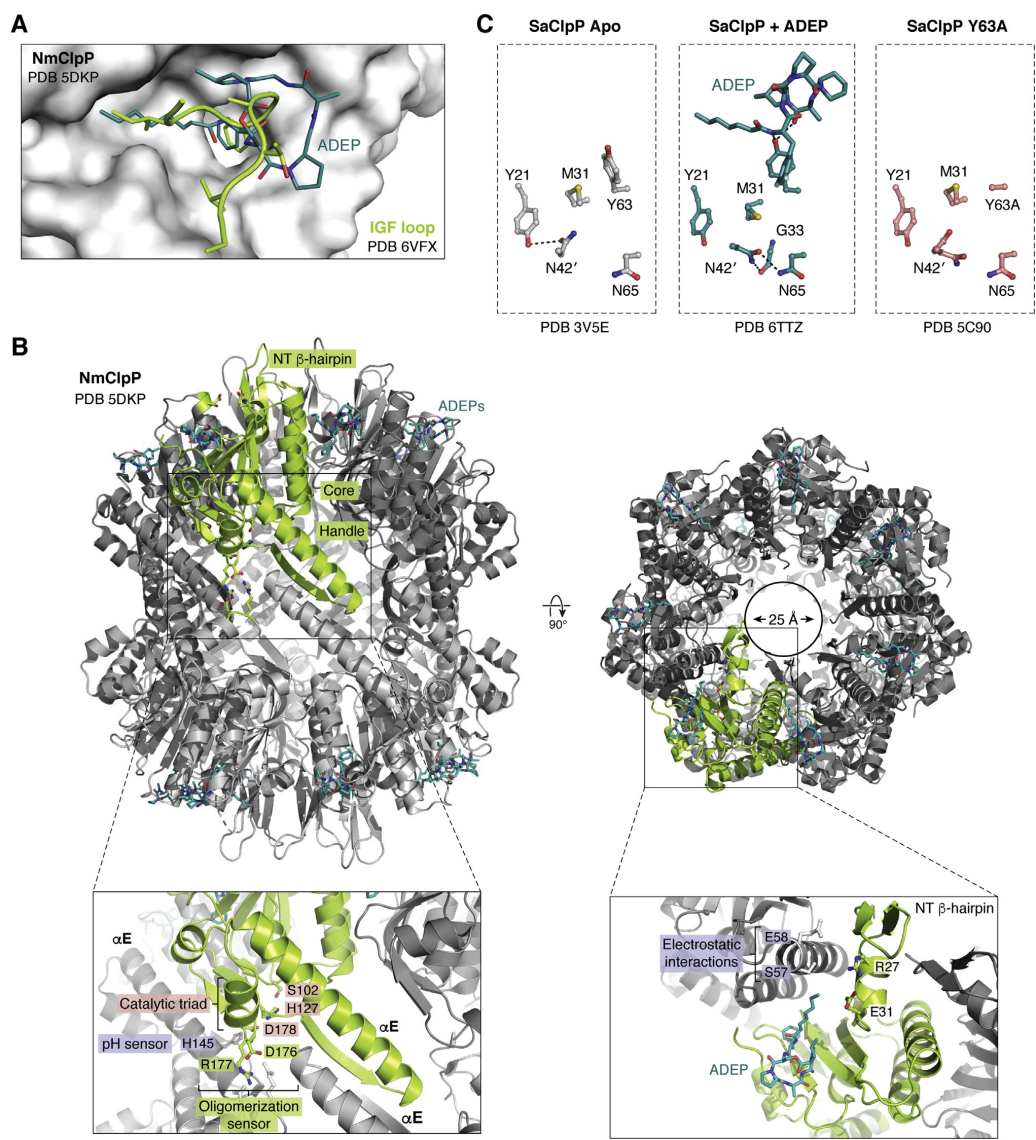
**Allosteric regulation of ClpP by small molecules and gain-of-function mutations.***A*, Acyldepsipeptide (ADEP, *teal sticks*) binds to H sites where the IGF loop (*light green cartoon*) of ATPases bind in the native complex, causing ClpP activation. The PDB IDs for the structures are 5DKP (NmClpP) and 6VFX (IGF loop). *B*, ADEP binding to NmClpP causes structural changes characteristic of activated complexes. ADEP binding to H sites causes the ordering of N-terminal loops into β-hairpin structures that *line* the opened axial pores (*upper right image*). ADEP binding causes the rigidification of the handle domains and the formation of interactions important for oligomerization, resulting in selection of the fully extended conformation. A rigidified handle domain consists of a β-strand followed by the long helix αE. (*Lower left box*) The catalytic triad (S102, H127, D178) is found in the core domain, close to the oligomeric sensor (OS) residues (D176, R177) and to the handle domain. Structural perturbations in the OS and handle domains distort the catalytic triad and the tetradecamer’s global conformation. A sensor residue, H145, found on the αE helix and close to the catalytic triad, mediates the pH-dependent conformational changes of NmClpP. (*Lower right box*) An electrostatic interaction network formed by S57 and E58 of one subunit and R27 and E31 of an adjacent subunit stabilizes the inactive closed gate NmClpP conformation. Binding of ADEP disrupts and remodels this network, resulting in β-hairpin formation of the N-terminal residues that leads to axial pore opening. The PDB ID for the structure is 5DKP. *C*, the SaClpP Y63A mutant is constitutively active and has an active open gate conformation. (*Left and middle*) ADEP binding to wild-type SaClpP (*green sticks*) causes a 90° rotation of the Y63 side chain that results in a downward domino effect on nearby residue M31 and N42 of a neighboring subunit (N42′) to avoid steric clash (PDB ID 6TTZ). The domino fall of M31 and N42′ (*middle*) engenders new electrostatic interactions with G33 and N65 and is proposed to cause axial pore opening seen in the crystal structure. *Gray sticks* (*left*) represent the starting wild-type SaClpP structure (PDB ID 3V5E) before ADEP binding. (*Right image*) The SaClpP Y63A mutant is constitutively active, and its crystal structure shows a similar domino effect in that N42′ exhibits one of two conformations (*pink stick* residues, PDB ID 5C90). The SaClpP Y63A + N42A double mutant is more active than the Y63A mutant. CryoEM class averages of the double mutant also show widened axial pores. ADEP, acyldepsipeptide; CryoEM, cryoelectron microscopy.

Our previous understanding of ClpP allostery has been gained largely from studies involving small-molecule agonists that can activate ClpP by binding to allosteric H sites and competing with the binding of the ATPase (76). The most studied compounds are ADEPs that have affinities in the low μM to sub-μM range for ClpP (Fig. 6*A*) (46, 59, 80, 133). Since the ClpX-ClpP interaction is highly dynamic and may involve ClpX ring rotation, a single ADEP molecule bound to an H site can cause rapid ClpX dissociation due to steric clash with moving IGF loops (dynamic competition), inhibiting substrate degradation (94). This inhibitory effect has been demonstrated across many species *in vitro* (76, 79, 90, 93, 94, 134, 135). At higher ADEP concentrations, however, ClpP activity is regained due to formation of the SMAC complex having more than one ADEP-bound H site (60).

Structures of SMACs continue to refine our understanding of ClpP allostery and have given rise to better agonists with promising clinical potential. There are at least three structural properties of SMAC revealed in studies using ADEPs: oligomerization/tetradecamerization, axial pore opening, and selection of the extended conformation (Fig. 6*B*). Full ClpP activation is achieved when all three structural characteristics are met, although partial activation can occur with less structural organization, creating an activation gradient for SMACs (45). Each of these structural elements induced by small molecule binding is discussed below.

### Oligomerization

Formation of the ClpP tetradecamer precedes the existence of the other two structural elements of SMAC, as no further organization is possible without complex assembly, and a ClpP monomer is catalytically inactive (Fig. 6*B*). ADEP binding to H sites, formed by two adjacent subunits, induces the tetradecamerization of monomeric *B. subtilis* ClpP (BsClpP) and heptameric human ClpP (HsClpP) (76, 91, 134, 136). Thus, ADEP binding stabilizes not only the interface of two subunits in a ring to form a heptamer, but also that of two ClpP rings to form the tetradecamer. ADEP binding also increases the thermostability of the ClpP tetradecamer (90).

### Axial pore opening

For protein substrates to be degraded, they must gain entrance into the ClpP chamber. As described earlier, ClpP has disordered N-terminal loops that plug the axial pores (83, 91). ADEP binding to H sites causes the ordering of these loops into β-hairpin structures (Fig. 6*B*, top left image). In this, the aliphatic chain of ADEP acts as a hydrophobic nucleus that initiates β-hairpin formation of the N-terminal residues by reorganizing electrostatic interactions near the axial pores. This is followed by retraction of these formed N-terminal β-hairpins away from the ClpP central axis, resulting in an opened pore (∼25–30 Å) (Fig. 6*B*, top right image) (83, 91). The seven β-hairpins that form the rim of each pore are held in place by extensive hydrophobic interactions with the core apical domain of ClpP. In NmClpP, the electrostatic network near the pores involves conserved residues, including R27, E31, S57, and E58 (Fig. 6*B*, lower right box). The combined mutation of E31A and E58A, which mimics ADEP binding by breaking the S57-E31 and R27-E58 interactions, results in a constitutively active NmClpP (83). The double mutant’s crystal structure shows widened axial pores, and methyl-TROSY NMR provides evidence for significant ordering of the N-terminal loops. It has been proposed that mere breaking of the said interactions may constitute the minimum requirement for ClpP activation, as it can result in axial pore opening and other important structural effects discussed below (83).

The importance of H sites in the allosteric control of ClpP is further highlighted in a gain-of-function mutant of *Staphylococcus aureus* ClpP (SaClpP). In the crystal structure of ADEP-bound SaClpP, the side chain of Y63, located at the H site, undergoes a 90⁰ rotation relative to its orientation in the inactive apoenzyme (Fig. 6*C*, left and middle panels). This rotation causes a domino effect to nearby subunits in avoidance of steric clashes and is presumed to result in N-terminal loop ordering and axial pore widening (137, 138). Mutation of Y63 to alanine partially recapitulates the structural effect of ADEP binding, as seen in a crystal structure of the mutant (Fig. 6*C*, right panel) (138). The mutant also degrades FtsZ *in vivo* and inhibits the growth of *S. aureus*. Additional mutation of the domino residue N42 (of an adjacent subunit, thus N42′) to alanine results in further SaClpP activation *in vivo* with the observed inhibition of cell division and sensitization to rifampicin. The cryoEM structure of the double mutant also showed widened axial pores (138).

### Selection of the extended form

**Figure 7:**
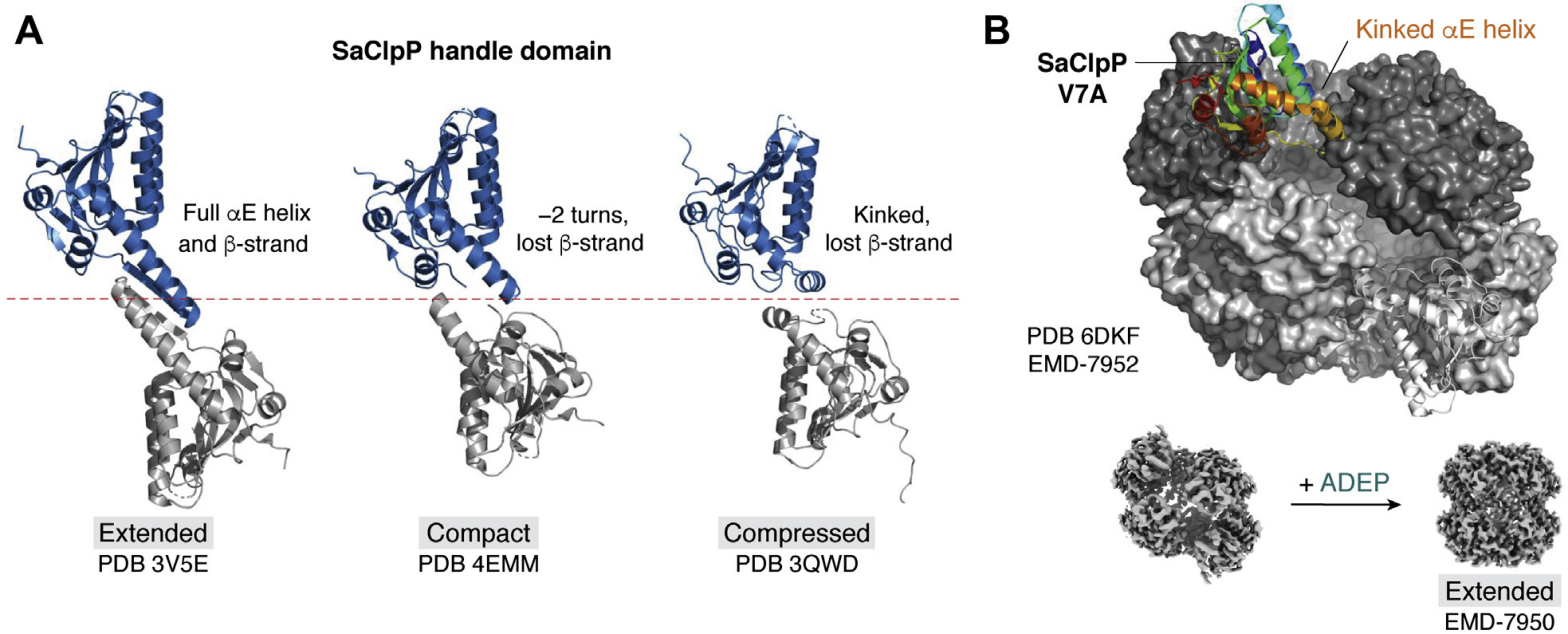
**The handle domain and N-terminal loops as allosteric sites of ClpP.***A*, the handle domain of ClpP controls the transition between extended and compact conformations. In the active, extended conformation of SaClpP (PDB ID 3V5E), a fully formed handle domain consists of a β-strand followed by a long helix αE. In the compact conformation, the αE helix loses 2 helical turns and the β-strand becomes disordered (PDB ID 4EMM). This perturbs the oligomeric sensor interactions and the catalytic triad geometry, resulting in an inactive ClpP. Further compression causes a kink in the αE helix, which breaks into two smaller helices, resulting in an inactive enzyme (PDB ID 3QWD). *B*, the N-terminus of SaClpP acts as a reversible conformational switch. Mutation of V7 to alanine results in a split-ring/lock washer conformation and an inactive enzyme. In this conformation, two opposing ClpP subunits have kinked αE helices, resulting in a large side opening in the tetradecamer (PDB ID 6DKF, EMD-7952). Addition of ADEP switches SaClpP V7A to the active, extended conformation (EMD-7950). ClpP, caseinolytic protease P; SaClpP, *Staphylococcus aureus* ClpP protease.

ClpP tetradecamerization relies on the interdigitation of handle domains, each of which consists of a β-strand structure and a long αE helix (Figs. 6*B* and 7*A*). The tetradecamer is further stabilized by the mutual interaction of oligomerization sensor (OS) residues (D176 and R177 in NmClpP) located in the core domain of ClpP (Fig. 6*B*, lower left box). Since the catalytic triad is also found in the core domain, in proximity with the OS and handle domains, ClpP activity is affected by structural disruptions in these domains. In fact, the active form of ClpP is associated with the fully extended structure in which the handle domains and OS interactions are intact as they help maintain proper catalytic triad geometry (Fig. 6*B*, lower left box). It follows then that compaction and compression of the ClpP tetradecamer can cause bending of the handle domains and distortion of catalytic triads, resulting in an inactive ClpP (Fig. 7*A*) (12).

ADEP binding has been shown to activate ClpP by stabilizing the extended form (46, 133). This allosteric effect originates from the surface H sites and travels ∼50 Å toward the ring–ring interface where the handle domains and OS residues are found (Fig. 6*B*, top right image). The extended form is stabilized upon rigidification of the handle domain and establishment of OS interactions. Handle domain rigidification upon ADEP/activator binding is observed in many crystal structures and supported by various biochemical and biophysical experiments, including methyl-TROSY NMR spectroscopy studies of NmClpP with a ^13^C spin label at a handle domain residue (I144). Similar results were observed in the activated NmClpP double mutant E31A + E58A (12, 83, 89). The ability of ADEPs to regain/retain the extended conformation is further demonstrated in the reactivation of the catalytic triad mutant, SaClpP D172N, which is compact in solution based on SAXS (90).

### A nuanced understanding of conformational selection and new layers of regulation

Recent studies have revealed additional layers of ClpP regulation that reflect the enzyme’s rugged energy landscape. First, a reversible, N-terminal conformational switch has been discovered through investigations of the SaClpP V7A mutant that exists predominantly in an inactive, lock-washer conformation (Fig. 7*B*) (139). This unusual structure is assembled by 12 SaClpP subunits in extended conformation and two opposing subunits in the rings in compressed conformation. As a result, large side pores are visible in the cryoEM structure of the complex. ADEP binding to this N-terminal mutant recovers its enzyme activity by conversion to the extended conformation as shown in another cryoEM structure (Fig. 7*B*, lower images).

Second, for NmClpP, pH-activity profiles have identified a pH-dependent conformational switch located at the handle region (140). This switch governs the equilibrium between inactive and active conformations in solution, with a pKa of 7.4 corresponding to the ionization of the handle domain residue, H145, that is closely associated with the catalytic triad (Fig. 6*B*, lower left box). At pH 7.0, NmClpP is predominantly in a compact conformation, while at pH 8.5, it is predominantly in the extended conformation, based on cryoEM structures for both species. At physiological pH of 7.4, the two conformational states coexist. Thus, handle domain residue H145 mediates the interconversion between conformations in response to small changes in pH.

Finally, substrate-binding pockets have been revealed as additional allosteric sites through elegant studies involving the *T. thermophilus* ClpP (TtClpP) and the *Mycobacterium tuberculosis* ClpP1P2 (MtClpP1P2) complexes (141, 142). Actinobacteria, including *M. tuberculosis*, are unique in that they harbor two ClpP isoforms, ClpP1 and ClpP2, that are inactive on their own as homo-oligomers, but can assemble into the active heterocomplex containing MtClpP1 and MtClpP2 heptameric rings (16, 143). The additional layer of regulation above is especially interesting given its counterintuitive nature. Compounds that bind to the enzyme active sites usually inhibit rather than activate the protease. Moreover, unlike the H sites that exert their allosteric effects from the apical surface of ClpP, this new allosteric site propagates its effect from the center, similar to the pH-dependent conformational switch located near the ring interface.

**Figure 8:**
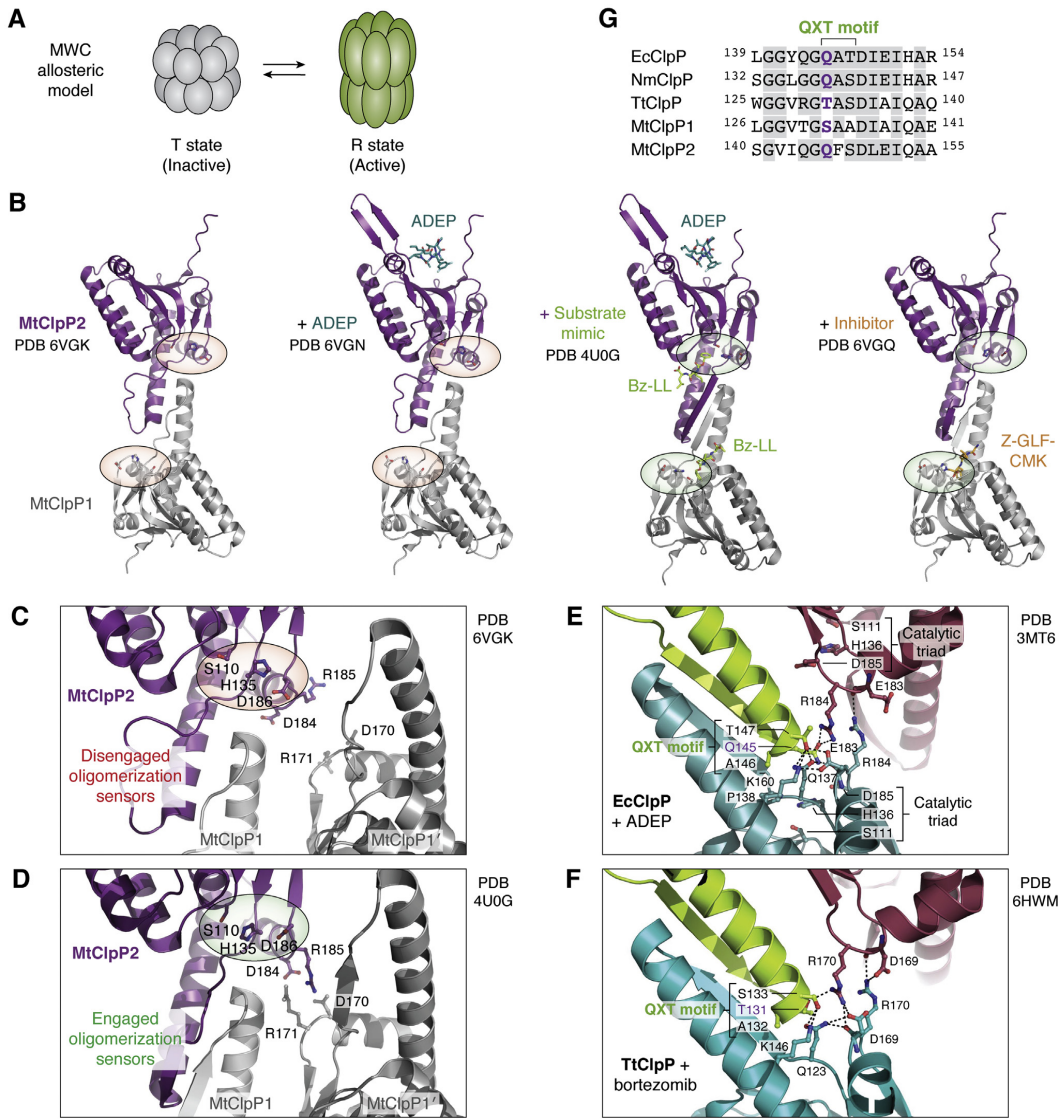
**Equilibria between inactive (T) and active (R) states of ClpP.***A*, a modified Monod-Wyman-Changeux (MWC) model is proposed for ClpP, in which an equilibrium exists in solution between inactive T state and active R state. The transition from the inactive to active conformation is highly cooperative and consists of changes in both intraring and interring interactions. *B*, the substrate binding site of some ClpPs are additional allosteric sites. For MtClpP1P2, the equilibrium is highly skewed towards the T state. Homo-tetradecameric rings of MtClpP1 or MtClpP2 are inactive, and only the MtClpP1P2 tetradecamer can be active (16, 143, 144). The inactive T state of MtClpP1P2 predominates in solution, as shown by the disordered catalytic triads of MtClpP1 and MtClpP2 (*red ovals*), due to disordered handle domains and oligomerization sensor interactions (PDB ID 6VGK). Only two ClpP subunits from opposing MtClpP1 and MtClpP2 rings are shown for clarity (first structure from *left*). Addition of ADEP, which binds exclusively to the MtClpP2 ring, is insufficient to fully form the handle domains and correct the catalytic triad geometry, although the axial pore loops/β-hairpins become ordered (second structure) (PDB ID 6VGN). Addition of the substrate mimic, benzoyl-leucyl-leucine (Bz-LL), results in organization of the handle domain and oligomeric sensor interactions, leading to correct catalytic triad geometries (*green ovals*) (third structure) (PDB ID 4U0G). Addition of the covalent inhibitor *Z*-Gly-Leu-Phe-chloromethyl ketone (Z-GLF-CMK) that specifically targets the MtClpP1 catalytic serine residue is sufficient to cause handle domain and catalytic triad organization (fourth structure) (PDB ID 6VGQ). *C*, the oligomeric sensor of MtClpP1P2 is important for complex activity. In the inactive T state, the oligomeric sensors of MtClpP1 and MtClpP2 rings are disengaged, distorting the nearby catalytic triad (*red ovals*) (PDB ID 6VGK). *D*, in the active R state, the oligomeric sensor residues engage and the catalytic triad geometry is corrected (*green ovals*) (PDB ID 4U0G). *E*, the equilibria between the T and R states of ClpP vary across species. This is proposed to arise from small sequence variations in the QXT motif. For EcClpP, the active R state predominates in solution. The QXT motif of EcClpP consists of Q145, A146, and T147. In the EcClpP-ADEP complex structure, this sequence forms a network of interactions with nearby residues at the oligomerization interface and very near the catalytic residues (*green ovals*) (PDB ID 3MT6). The interaction network includes the oligomerization sensor residue R184, and Q137 of the conserved HQP motif (H136, Q137, P138). Residue H136 of the HQP motif is part of the catalytic triad. These interactions stabilize the R state over the T state of EcClpP. *F*, for TtClpP, the QXT motif has the sequence TAS, which, compared to the QAT sequence of EcClpP, forms fewer stabilizing interactions to support the extended conformation. In the presence of bortezomib at substoichiometric concentrations, the oligomerization sensor residues are engaged, leading to the stabilization of the active R state (PDB ID 6HWM). The potential of TtClpP’s TAS sequence to stabilize the R state is less than that of EcClpP’s QAT sequence. This skews the equilibrium to the T state for TtClpP. *G*, the QXT motifs of EcClpP, NmClpP, TtClpP, MtClpP1, and MtClpP2 diverge in sequence. This might partly explain the differential equilibria between T and R states in solution. For MtClpP1, the sequence for this motif is drastically changed to SAA relative to EcClpP’s QAT, so that only upon complexation with MtClpP2 (containing the QFS sequence) can an active complex be achieved. MtClpP1P2 is therefore predominantly in the T state in solution, even more so than TtClpP. ClpP, caseinolytic protease P; EcClpP, *Escherichia coli* ClpP protease; MtClpP, *Mycobacterium tuberculosis* ClpP protease; NmClpP, *Neisseria meningitidis* ClpP protease; QXT, glutamine-any amino acid X-threonine.

Compared with EcClpP and others, TtClpP is predominantly in an inactive state in solution, and MtClpP1P2 even more so. The relationship between ClpP conformers in these two species is described by a modified Monod–Wyman–Changeux (MWC) model, depicting an equilibrium that strongly favors the inactive, compact T state over the active, extended R state (Fig. 8*A*). For MtClpP1P2, the equilibrium is much more heavily skewed toward the T state than for TtClpP (141). Even with the addition of ADEP to MtClpP1P2, which binds exclusively to the MtClpP2 ring, only minimal activation is achieved due to incomplete organization of the handle domains and OS interactions that fail to sustain a proper catalytic triad (Fig. 8, *B* and *C*) (79, 135, 141). Interestingly, the addition of substrate mimics such as benzoyl-leucyl-leucine (Bz-LL) or benzyloxycarbonyl-Gly-Leu-Phe-chloromethyl ketone (Z-GLF-CMK, an MtClpP1-specific, catalytic serine covalent inhibitor) at substoichiometric concentrations results in a highly cooperative transition toward the R state, mediated by both intraring and interring allosteric effects (141, 144). In the R state, the handle domains and OS interactions are fully formed, and the catalytic triads have correct geometry (Fig. 8, *B* and *D*) (141). This phenomenon suggests substrate regulation in MtClpP1P2 that can be exploited in drug development by specifically targeting the MtClpP1P2 T state. A similar, highly cooperative T to R transition has been observed for TtClpP upon addition of the dipeptide substrate mimic, bortezomib (142).

The above studies on MtClpP1P2 and TtClpP using activating substrate mimics raise an interesting observation in that ClpP from different species appears to have varying degrees of regulation. For instance, compared with EcClpP, MtClpP1P2 is more strictly regulated, such that full activation is possible only with the assembly of both MtClpP1 and MtClpP2 rings, and in complex with an ATPase (ClpC1 or ClpX) or in the presence of a bound peptide (or mimic) (17, 18, 141, 143, 144). TtClpP, although less tightly regulated than MtClpP1P2, is nonetheless less active in solution than EcClpP under the same conditions (142). This is quite intriguing for highly conserved enzymes such as ClpP. It has been suggested that these functional differences are coded in sequence variations within the QXT motif found at the tip of handle domains and directly interacting with OS and catalytic triad residues (142). In EcClpP, the QAT sequence is better able to sustain the extended conformation through a network of noncovalent interactions at the ring interface (Fig. 8*E*). The corresponding TAS sequence in TtClpP leads to fewer interactions, and therefore, a less sturdy chamber that needs extra structural support from substrate binding at one or more catalytic sites (Fig. 8*F*). For MtClpP1, the changes are even more drastic with the SAA sequence, but MtClpP2 bears a more conserved sequence, QFS (Fig. 8*G*). Nevertheless, the importance of the QXT motif in ClpP function needs further validation by mutagenesis and structural studies. Given the need for *M. tuberculosis* populations to maintain cellular proteostasis during dormancy, it is not surprising that such tight regulation of MtClpP1P2 has evolved in this organism (8, 145).

## Concluding remarks

CryoEM structures of ClpP-ATPases from different organisms have illuminated the structural elements that enable the productive interaction between their two asymmetric components. The dynamic interaction is mediated by conserved loops that facilitate docking of ATPase on ClpP and support the conformational changes needed for substrate unfolding and translocation. Possible mechanisms for substrate translocation have been presented based on available structural and biochemical data, although certain aspects of the mechanisms need more evidence. Studies using small-molecule modulators of ClpP function have also presented new insights into the enzyme’s allostery, revealing a highly dynamic chamber in equilibrium between active and inactive states, and whose function is controllable at several allosteric sites. Given the significance of ClpP-ATPase complexes in cellular health and survival in bacterial pathogenesis and in cancer, a fuller understanding of their structure, function, and behavior will be beneficial to current drug development efforts that target this proteolytic complex.

## Conflict of interest

The authors declare that there is no conflict of interest with the contents of this article.
